# Supplementary material for: From Early Diagnoses to New Treatments for Liver, Pancreatic, Gastric, and Colorectal Cancers Using Carbon Nanotubes: New Chances Still Underexplored
Source: Int J Mol Sci. 2025 Sep 20;26(18):9201. doi: 10.3390/ijms26189201 (PMC12471133; doi:10.3390/ijms26189201)
Supplement: Supplementary file 1 [file ijms-26-09201-s001.zip › ijms-3862556-supplementary.pdf]

Supplementary Materials

From Early Diagnosis to New Treatments for Liver, Pancreatic, Gastric and Colorectal Cancers Using Carbon Nanotubes: New Chances Still Too Little Explored

Silvana Alfei [1,*], Caterina Reggio [2] and Guendalina Zuccari [1,2,*]

[1] Department of Pharmacy (DIFAR), University of Genoa, Viale Cembrano, 4, 16148 Genoa, Italy
[2] Laboratory of Experimental Therapies in Oncology, IRCCS Istituto Giannina Gaslini, Via G. Gaslini 5, 16147 Genoa, Italy; caterinareggio@gaslini.org
* Correspondence: alfei@difar.unige.it; Tel.: +39 010 355 2296 (S.A.); guendalina.zuccari@unige.it (G.Z.)

Additional Tables

Table S1. Available experimentation using CNTs-based sensors to detect biomolecular markers of the most reported cancers.

Cancer Marker	Types of biosensors	Nano sensor	LR/LOD
AKT2 gene *	TFT-based-GSs	CNT	LOD = 2 fM
miRNA-21 *	FET-GSs	CNT/Au	LR = 1 aM-1nM, LOD = 0.87 aM
T47D cancer cells	TFT	PB- CNT-DNA	LR = 1.6×10^{-4}-5 mmol/L, LOD = 0.88 mg/L
DNA	ECs	CNT/Au	LOD = 5.2 fM
Let-7a miRNA	PECSs	BiOI-BSCPC/CNT	LR = 0.2–2000 pM, LOD = 0.1 pM
CPT-11	EC	dsDNA/poly (CTAB-MWCNTs)	LR = 2–10 μg/mL and 10–500 μg/mL, LOD = 1.03 μg/mL
5-hmC-ds DNA	CLSs	PDMDAAC /MWCNTs	LR = 1.0×10^{-11}-2.0×10^{-9} M, LOD = 2.3×10^{-12} M
Cystatin C	EC	P-Py/MWCNT	LR = 0–300 ng/mL, LOD = 28 ng/mL
CEA (CC)	ECISs	Au/CNT	LR = 0.005–50 ng/mL
VEGF 165 (MAGG)	ECASs	PANI/CNT	LR = 0.5 pg/mL-1 μg/mL, LOD = 0.4 pg/mL
CA125 (OC)	ECISs	MOF-808/CNT	LR = 0.1–0.1–30 ng/mL, LOD = 0.5 pg/mL
DNA *	EG-CNT FET	CNT	LR = 10 fM-10 pM, LOD = 0.21 aM
DNA *	TFT biosensor	CNT	LR = 10 pM-1 μM, LOD = 42 fM

Copyright by Elsevier (2024). Reproduced under license order number 501996892 and license number 6055460280340, dated 25 June 2025 (https://s100.copyright.com/CustomerAdmin/PLF.jsp?ref=858e9ed9-c41d-4986-92f1-0e15a44a845d, accessed on 25 June 2025). *Breast cancer; LR = linear range; LOD = limit of detection; PB = peptide-based; TFT = thin-film-transistor; FET = field-effect transistor; CTAB = cetyl trimethylammonium bromide; Au = gold; MOF = metal organic framework; MOF-808 = Zr-trimesic acid MOF; MWCNTs = multiwalled carbon nanotubes; CEA = carcinoembryonic antigen; CC = colorectal cancer; MAGG = marker of angiogenesis; OC = ovarian cancer; GSs = gene sensors; ECSs = electrochemical sensors; ECISs = electrochemical immune sensors; PECSs = photoelectrochemical sensors; EG = electrolyte-gated; ECASs = electrochemical-aptasensor; CLSs = chemiluminescence sensors; BSCPC = Bi-(semiconductor photocatalysts); *ds* = double strain; PDMDAAC = poly-(dimethyl diallyl ammonium chloride); P-Py = poly-pyrrole; PANI = polyaniline.

Table S2. Clinical trials assessing the possible application of CNTs in anticancer therapy.

Trial Number	Sponsor	Device/Intervention	Prob-lem	Partici-pants	Study Sta-tus	Ending year	Ref
NCT01773850 *	UNC Lineberger Comprehensive Cancer Center	CNT x-ray source array for SDT of BN	BN	54	Completed	2018	[180]
NCT01420588 **	Anhui Medical University	*Of*-AUNPs/CNTs-based CNSs	PCGLs	1000	Completed	2020	[181,182]

SDT = Stationary digital tomosynthesis; BN = breast neoplasm; PCGLs = pre-cancerous gastric lesions; AuNPs = gold nanoparticles; CNTs = carbon nanotubes: *Of* = organically functionalized; CNSs =chemical nanosensors; * study title: Stationary carbon nanotube X-ray digital breast tomosynthesis scanner; ** study title: Diagnosis of gastric lesions from exhaled breath and saliva. References in this Table correspond to that reported in the main text, while information has been found in the reference cited as ref. [5] in the main text.

Table S3. Study type assessment.

Trial Number	Sponsor	Study Type	Ethics Approval	Status	Ref
NCT01773850 *	UNC Lineberger Comprehensive Cancer Center	OCPS	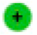	Completed	[180]
NCT01420588 **	Anhui Medical University	OCPS	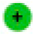	Completed	[181,182]

OCPS = Observational cohort prospective study * study title: Stationary carbon nanotube X-ray digital breast tomosynthesis scanner; ** study title: Diagnosis of gastric lesions from exhaled breath and saliva; 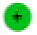 = positive ethical approval. References in this Table correspond to that reported in the main text, while information has been found in the reference cited as ref. [5] in the main text.
